# Analysis of Clinical, Pathological and Prognostic Features of Coexistent Membranous and IgA Nephropathy in a Series of 13 Patients at a Tertiary Care Hospital

**DOI:** 10.7759/cureus.18006

**Published:** 2021-09-15

**Authors:** Nida Saleem, Saima Bashir, Syed Nayer Mahmud, Muhammad Haneef, Humaira Nasir, Danish Jabbar

**Affiliations:** 1 Nephrology, Shifa International Hospital, Islamabad, PAK; 2 Histopathology, Shifa International Hospital, Islamabad, PAK

**Keywords:** overlapping glomerulonephritis, pla2r, renal biopsy, iga nephropathy, membranous nephropathy

## Abstract

Background

Membranous nephropathy (MN) and immunoglobulin A nephropathy (IgAN) are although two different entities, yet can rarely coexist. There is not much data available on this coexistent disorder, specifically with regard to the treatment modality and outcome. Here, we analyse in detail, retrospectively, 13 cases of coexistent IgA and membranous nephropathy (IgA-MN).

Methods

Renal biopsy data of 1084 diagnosed cases of either membranous or IgA nephropathy was obtained from March 2015 till March 2021. Out of 1084 patients, 19 diagnosed cases of the coexistent disorder were identified. Six out of 19 patients were excluded because of their unwillingness. From remaining 13 patients, data regarding clinical presentation, investigations, management and treatment response was collected from hospital database, files and via telephonic interview.

Results

The overall prevalence noted was 1.75%. Among them, 53.8% were females and 46.2% were males. Their median age was 40 years (range: 14-71 years). On workup, mean serum albumin was 2.64 g/dl (range: 1.6-3.8 g/dl), mean proteinuria was 5.5 g/24 hours (range: 1.55-11.48 g/24 hours) and mean creatinine was 0.98 mg/dl (range: 0.5-2.8 mg/dl). Anti-phospholipase A2 receptor antibody positivity was only 14.2%. The renal biopsy of all patients showed thickening of the glomerular basement membrane with granular IgG deposits and mesangial expansion with granular IgA deposits. A total of 80% patients showed complete remission with steroids, calcineurin inhibitors (CNIs) and angiotensin-converting enzyme inhibitors/angiotensin receptor blockers (ACEi/ARBs).

Conclusion

IgA-MN is probably a separate disorder that can only be confirmed on immunofluorescence microscopy. The response to the combination of steroids, CNIs and ACEi/ARBs is found to be the most effective; hence, this combination must often be used for the management of this coexistent disorder.

## Introduction

Immunoglobulin A nephropathy (IgAN) and membranous nephropathy (MN) are two separate immune-complex-mediated glomerular disorders that are characterized by different clinical manifestations, pathogenic mechanisms and histopathological findings and are managed in accordance with different treatment guidelines. Although the prevalence of coexistent IgA and membranous nephropathy (IgA-MN) is very rare, yet, it has been suggested that this disorder is more common in the Chinese population [1].

IgAN is the most common glomerular disorder worldwide [2-4]. It usually presents with asymptomatic hematuria, sub-nephrotic proteinuria, hypertension and renal impairment in later stages. It is diagnosed on histopathology and immunofluorescence by characteristic IgA deposits in glomerular mesangium. MEST-C (mesangial and endocapillary hypercellularity, segmental sclerosis, interstitial fibrosis/tubular atrophy, and the presence of crescents) scoring of light microscopic findings can help in the prognostication of IgAN [5].

MN is the most common cause of nephrotic syndrome in adults [4]. In contrast to IgAN, it usually presents with nephrotic syndrome characterized by proteinuria greater than 3.5 g/24 hours, hypoalbuminemia, edema and hyperlipidemia. It can be diagnosed serologically by measuring antibody titers like anti-phospholipase A2 receptor (anti-PLA2R) antibodies, anti-thrombospondin antibody and many others. Furthermore, histopathological evaluation, by identifying characteristic sub-epithelial IgG dominant immune deposits in immunofluorescence and thickened glomerular basement membrane in most cases on light microscopy, plays a major role in diagnoses of this disorder. Because of the low incidence, only a few reports are found in the literature, focusing mainly on histopathological features [6-9].

Although the prevalence of this coexistent disorder is rare, it is still necessary to create awareness among physicians to think about this possibility in case of an atypical presentation of patients [3,7]. Furthermore, it is not well known whether this overlapping disorder is a distinct clinicopathological entity or has progressed from one to the other disorder [7,10]. In addition to this, there is no published data that has specifically described about the effectiveness of a particular treatment modality for this coexistent disorder.

The main aim of this case series is to gain a deeper understanding of this overlapping disorder and to determine presenting features, pathological findings, treatment received by these patients and response to different non-immunosuppressive and immunosuppressive treatment modalities.

## Materials and methods

Renal biopsy data of 1084 diagnosed cases of membranous and IgA nephropathy was obtained from March 2015 till March 2021. Out of these 1084 patients, 19 diagnosed cases of coexistent membranous and IgA nephropathy were included based on light microscopic findings of glomerular basement membrane thickening and mesangial expansion and immunofluorescence findings of mesangial IgA and glomerular basement membrane IgG including IgG4 immune complex deposits. Out of these 19 diagnosed cases of coexistent disorder, six patients were excluded based on either their unwillingness or inaccessibility. From these patients, data regarding clinical presentation, investigations and management and treatment response was collected retrospectively from hospital electronic database, files and via telephonic interview. Their medical records were reviewed for their age, gender, co-morbidities, family history, history of recent infection, serum creatinine, albumin, 24-hour urinary protein, immunological workup, treatment received and response to treatment in terms of no remission, partial remission and complete remission.

Complete remission was defined as the normalization of serum albumin to above 4 g/dl and reduction in proteinuria to less than 300 mg/24 hours. Partial remission was defined as 24-hour urinary protein between 300 mg/24 hours and 3.5 g/24 hours and serum albumin >3 g/dl or 50% reduction in proteinuria from baseline. No remission was defined as persistence of proteinuria >3.5 g/24 hours and serum albumin <3 g/dl despite immunosuppressive therapy.

We noted findings on both light and immunofluorescence microscopy of these 13 patients. We then determined the degree of glomerular basement membrane thickening, mesangial expansion, chronicity determined by glomerulosclerosis, interstitial fibrosis and tubular atrophy. Furthermore, two scoring systems were used to assess light microscopic findings of these patients. These include MEST-C and total renal chronicity score (TRCS). MEST-C scoring system includes five variables: mesangial hyper-cellularity (M), endocapillary hyper-cellularity (E), segmental sclerosis (S), tubular atrophy (T) and crescents (C). Each variable is scored based on the degree of involvement. Regarding the TRCS scoring system, scoring is done based on four variables that include glomerulosclerosis, interstitial fibrosis, tubular atrophy and arteriolosclerosis. According to this scoring system, a score of 0-1 is considered as minimal, 2-4 as mild, 5-7 as moderate and >8 as severe chronicity score.

After assessing medical records and taking detailed history, one out of five combinations of drugs was found to be taken by these 13 patients and included oral medication for a duration of six months. First group included angiotensin-converting enzyme inhibitors/angiotensin receptor blockers (ACEi/ ARBs) and spironolactone, second included ACEi/ARBs, steroids and spironolactone, third included ACEi/ARBs, spironolactone and calcineurin inhibitors (CNIs), fourth included ACEi/ARBs, steroids and CNIs and the final one included ACEi/ARBs, steroids and mycophenolate mofetil (MMF).

## Results

Clinical and serological features of these 13 patients are summarized in Table 1 below.

**Table 1:** Summary of clinical and laboratory features of 13 patients neg: negative, +ve: positive, ---: not done, N: normal, M: male, F: female, Hep C+: hepatits C positive, HTN: hypertension, NSAID: non-steroidal anti-inflammatory drug, BP: blood pressure, anti-PLA2R: anti-phospholipase A2 receptor, ANA: anti-nuclear antibody, C3: complement 3, ANCA: anti-nuclear cytoplasmic antibody, ASOT: anti-streptolysin O titer

	Case 1	Case 2	Case 3	Case 4	Case 5	Case 6	Case 7	Case 8	Case 9	Case 10	Case 11	Case 12	Case 13
Age (years)	22	51	15	41	35	30	53	40	14	51	47	71	20
Gender	F	M	F	M	F	F	M	F	F	M	F	M	M
Comorbids	No	Hep C+	No	No	HTN	HTN	NSAID intake	No	No	NSAID intake	No	HTN, NSAID intake	No
Family history of kidney disease	No	No	No	No	No	No	No	No	No	No	No	No	No
Recent infection history	No	No	No	No	No	No	No	No	No	Yes	No	No	No
Presenting complaint edema	+++	+++	++	+++	++++	+++	++	+++	+++	+++	++	+++	++
Raised BP	No	No	No	No	Yes	Yes	Yes	No	No	No	No	Yes	No
Serum creatinine (mg/dl)	0.7	0.77	0.59	0.6	2.8	0.9	0.8	0.65	0.5	0.8	0.9	2.21	0.6
Serum albumin (g/dl)	2.8	2.8	2.8	2.6	2.9	2.7	1.8	2.4	1.98	3	3.2	1.6	3.8
Urinary RBCs (HPF)	40	6-8	>50	4-6	20-30	2-3	Nil	3-4	0-1	4	---	10-15	10-15
Urinary protein (g/24 hours)	3.9	8.5	6	2.557	8.115	6.147	4.47	5.95	7	3	1.55	11.48	3.1
Serum cholesterol (mg/dl)	245	280	635	390	420	216	330	280	323	208	230	233	390
Anti-PLA2R level (RU/ml)	-----	+165	neg 1.3	---	neg 1.25	neg 2	neg	---	---	neg	neg	neg	---
ANA	neg	----	neg	neg	neg	---	+ve 9.6	neg	++	neg	neg	neg	neg
C3	N	----	N	N	N	---	N	N	N	N	N	N	----
Serum IgA level	-----	----	----	---	---	---	-----	---	--	3.68+	---	---	----
Hepatitis serology	neg	Hep C+	neg	neg	neg	neg	neg	neg	neg	neg	neg	neg	neg
ANCA profile	-----	-----	----	---	neg	----	---	---	neg	---	---	neg	----
ASOT	----	-----	neg	---	---	---	neg	neg	--	---	---	----	---

As seen in Table 1, the median age of these patients was 40 years (range: 14-71 years). Among these 13 patients, 53.8% were females and 46.2% were males. There was no family history of any renal disease. Only one patient had a history of sore throat one week before presentation. Three out 13 patients had a history of hypertension and non-steroidal anti-inflammatory drug (NSAID) use in the past. All patients presented with bilateral moderate to severe pedal edema. Post-presentation, four out of 13 patients had uncontrolled hypertension. There was no history of macroscopic hematuria at the time of presentation. Seven out of 13 patients had microscopic hematuria (>5 RBCs/HPF). All patients, except one, were hypoalbuminemic (serum albumin <3.5 g/dl). Mean serum albumin was 2.64 g/dl (range: 1.6-3.8 g/dl). A total of 11 out of 13 patients had nephrotic range proteinuria. Other two patients had proteinuria above 1 g/24 hours. Mean proteinuria was 5.5 g/24 hours (range: 1.55-11.48 g/24 hours). All patients had raised serum cholesterol levels (>200 mg/dl). The median serum total cholesterol level was 280 mg/dl (range: 208-635 mg/dl). Only two patients had raised serum creatinine. Mean creatinine of these 13 patient was 0.98 mg/dl/ (range: 0.5-2.8 mg/dl).

Regarding the serological workup, anti-PLA2R antibodies, calculated via the enzyme-linked immunosorbent assay (ELISA), were negative (<14 RU/ml) in seven out of 13 patients. One patient had PLA2R antibody positivity with a level of 165 RU/ml. No PLA2R antibody testing was done in the remaining five patients due to the unavailability of an anti-PLA2R assay at our setup before 2017. A total of 10 out of 13 patients had normal complement levels. Anti-nuclear antibody (ANA) came out to be positive in two out of 11 tests performed. One patient had hepatitis C infection. Anti-streptolysin O (ASO) titer and anti-nuclear cytoplasmic antibody (ANCA) serology were negative in three patients out of three tests being done. One patient had positive IgA antibodies out of only one test being performed. Histopathological features are described in Table 2.

**Table 2:** Summary of light microscopic findings Mod: moderate, GS: glomerulosclerosis, TRCS: total renal chronicity score, MEST-C: mesangial hyper-cellularity, endocapillary hyper-cellularity, segmental sclerosis, tubular atrophy, crescents

	Case 1	Case 2	Case 3	Case 4	Case 5	Case 6	Case 7	Case 8	Case 9	Case 10	Case 11	Case 12	Case 13
Thickening of GBM	Mod	Mild	Mild	Mild	Mild	Mod	Normal	Marked	mod	Mild	Mod	Mild	Normal
Degree of mesangial cellularity expansion	Mod	Mild	No	Mild	Mod	No	Mild	Mild	Diffuse	Mild	Mild	Mod	Minimal
Glomerular sclerosis	Yes	No	No	Yes	Yes	Yes	No	No	No	Yes	No	Yes	No
Interstitial fibrosis	Mild	Mild	No	No	Mild	Mild	No	No	No	No	No	No	No
Tubular atrophy	Mild	Mild	No	No	Mild	Mild	No	No	No	No	No	Mild	No
Crescent	Yes, 1 cellular	No	No	No	Yes, 1 fibrous	No	No	No	No	No	No	No	No
TRCS score	5	2	0	1	4	2	0	0	0	2	1	3	0
MEST-C score	5	2	0	2	4	2	1	1	1	2	1	3	1

**Figure 1:**
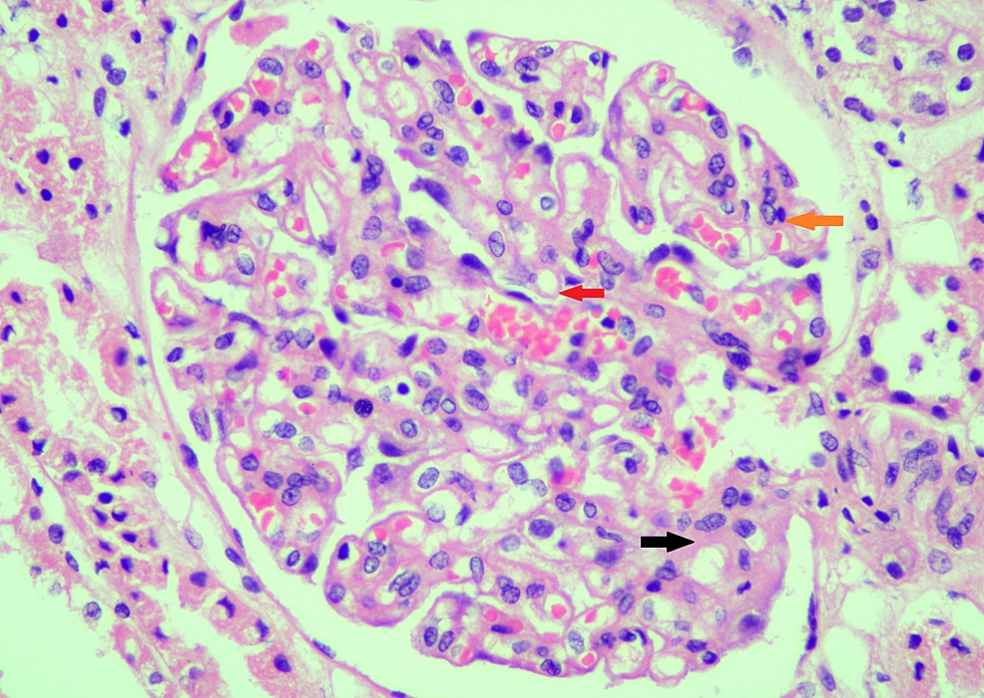
Light microscopic image (H and E staining, X40) Thickened glomerular basement membrane (red arrow); mild mesangial hyper-cellularity (orange arrow); mild focal mesangial expansion (black arrow)

**Figure 2:**
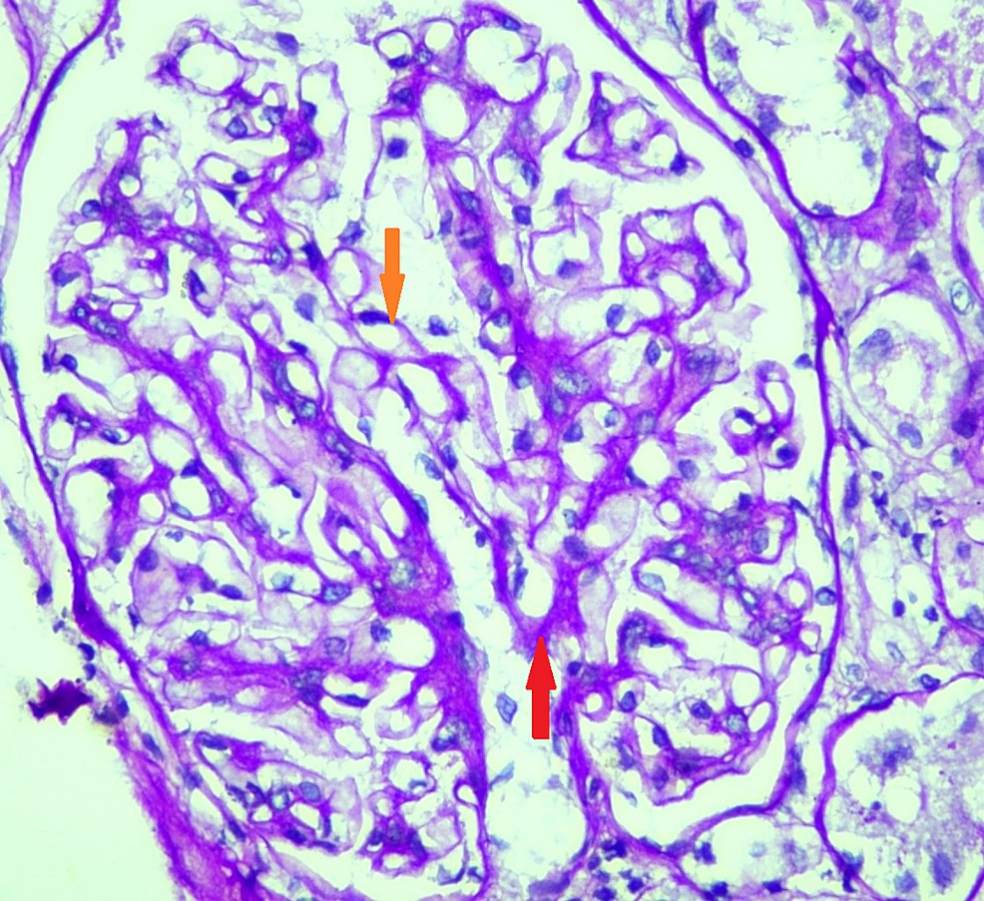
Light microscopic image (PAS staining, X40) Mild mesangial expansion (red arrow); thickened glomerular basement membrane (orange arrow). PAS: periodic acid–Schiff

As summarized in the table, light microscopic findings of all these 13 patients were compared based on the degree of glomerular basement membrane thickening, mesangial expansion and cellularity, presence of crescents, glomerulosclerosis, tubular atrophy and interstitial fibrosis. MEST-C and TRCS prognostic scoring systems of IgA and membranous nephropathy were applied and scores were calculated, respectively. Glomerular basement membrane thickening was normal in two patients, mild in six patients, moderate in four patients and marked in one patient. Only one patient, who was later diagnosed as a case of lupus nephritis, had diffuse mesangial expansion accompanying mesangial hyper-cellularity. Majority of patients had no to mild mesangial expansion (eight out of 13). Some degree of glomerulosclerosis either segmental or global was present in six out of 13 patients. Four had mild interstitial fibrosis and five had mild tubular atrophy. Remaining patients had neither interstitial fibrosis nor tubular atrophy. Only two patients had crescents: one fibrous and the other had cellular type. These two patients with crescentic glomerulonephritis had higher MEST-C and TRCS scores (4 and 5). Out of remaining 11 patients, seven had a minimal chronicity score and four had a mild score. Regarding the MEST-C score, out of 11 patients without crescents, one had 0 score, five had 1 score, four had 2 score and one had 3 score. Figures 1, 2 show light microscopic images of case 2 having positive PLA2R antibody titer.

These images show focally thickened glomerular basement membrane and mild mesangial hyper-cellularity with mesangial expansion. Immunofluorescence findings are summarized in Table 3.

**Table 3:** Summary of immunofluorescence findings C: case, GBM: glomerular basement membrane, ND: not done, C3: complement 3

Patient	IgG		IgA		IgM		C3		C1q		Others
	GBM	Mesangium	GBM	Mesangium	GBM	Mesangium	GBM	Mesangium	GBM	Mesangium	IgG4
C.1	+++	+/-	---	+++	+	+	++	++	--	--	++
C.2	+++	---	---	+++	---	+/-	+/-	---	+/-	+/-	++
C.3	+++	----	+/-	+++	++	++	++	+	+/-	+/-	+++
C.4	+++	---	---	+++	---	+/-	++	++	---	---	++
C.5	++	---	++	++	+/-	+	+	+	---	---	---
C.6	+++	----	+/-	++	+/-	+/-	+/-	----	---	---	++
C.7	+++	--	--	+++	+/-	+	--	---	---	+/-	---
C.8	+++	++	---	++/-	+/-	+	--	----	+/-	+/-	+++
C.9	+++	----	+	+/++	+/-	+	++	+	+	++	++
C.10	+++	----	----	++	+/-	--	+/-	---	----	+/-	+++
C.11	+++	----	----	++	+	+	+	----	---	---	+/-
C.12	+++	----	---	++	--	+/-	---	+/-	----	---	ND
C.13	+++	---	-----	+++	--	++/--	---	----	----	---	ND

**Figure 3:**
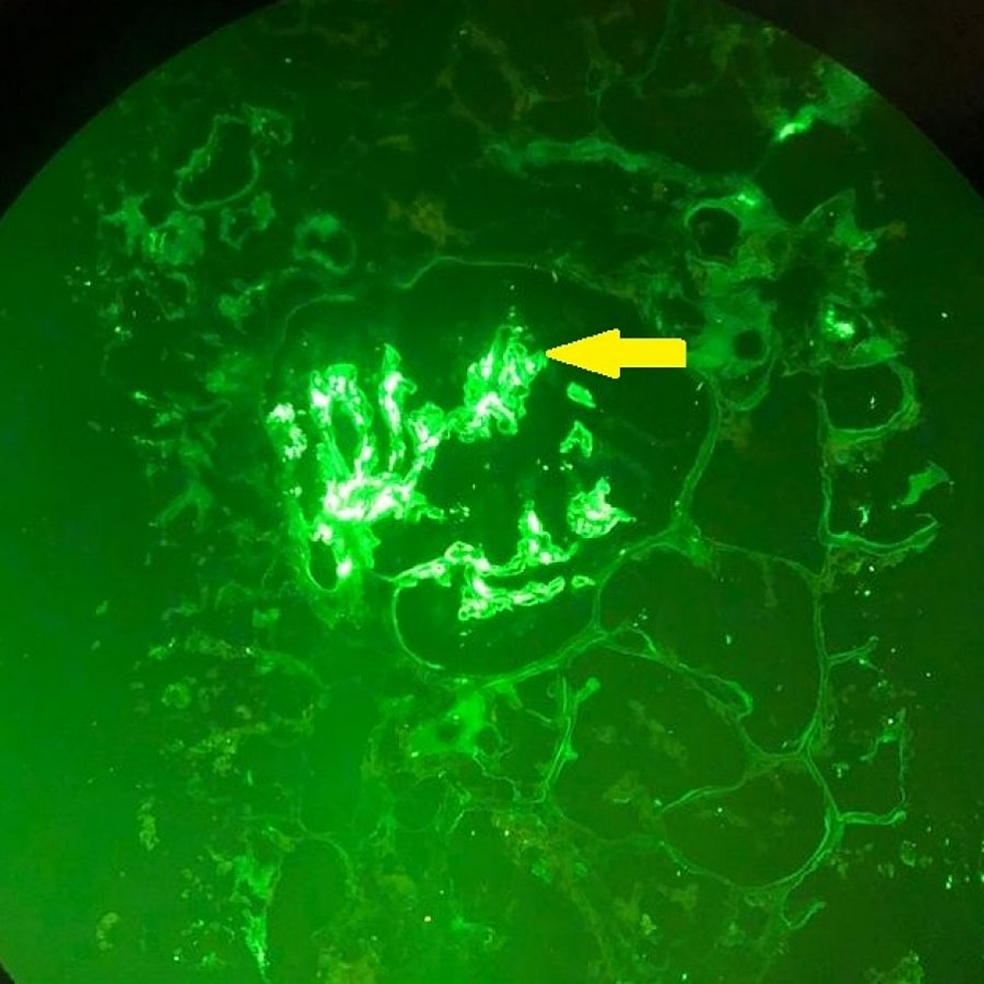
Immunofluorescence image (IgA staining, X40) Mesangial IgA deposits (yellow arrow)

**Figure 4:**
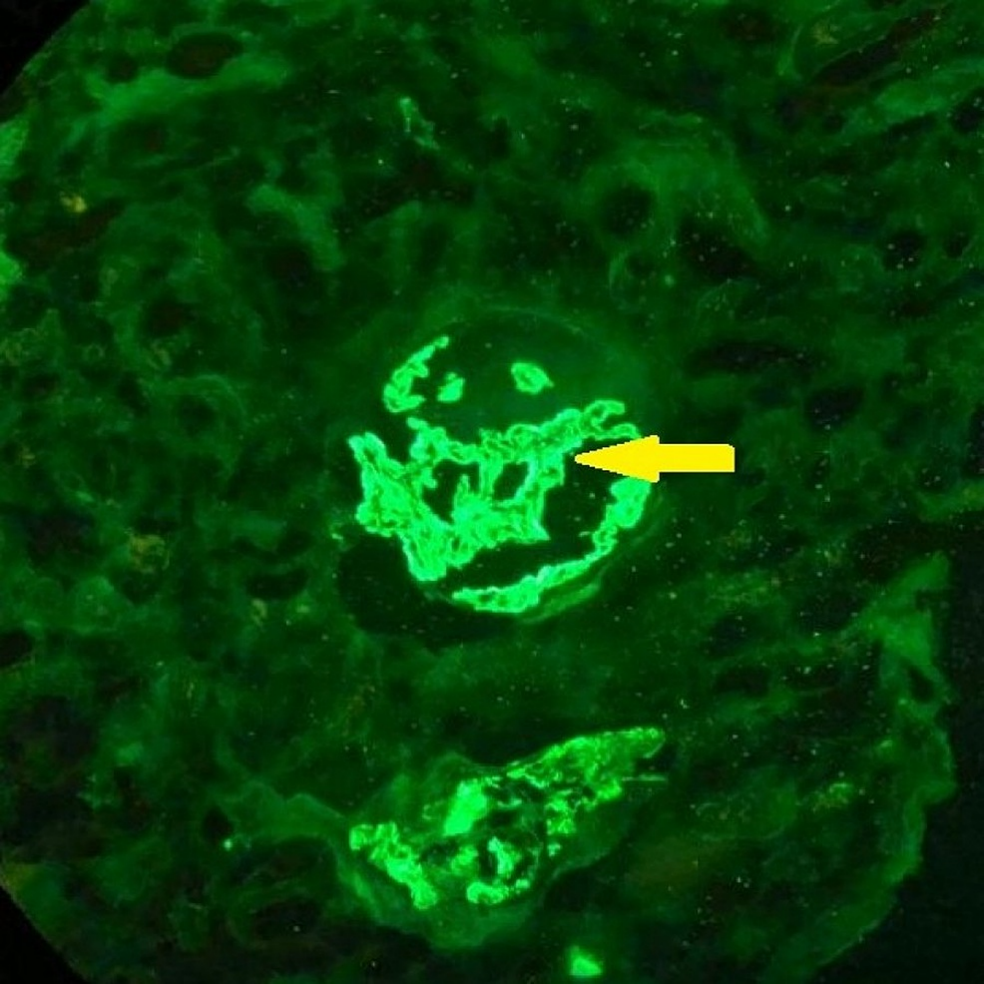
Immunofluorescence image (IgG staining X40) Membranous IgG deposits (yellow arrow)

These findings showed IgG-containing granular immune deposits in the glomerular basement membrane and moderate to marked IgA-containing granular immune deposits in the glomerular mesangium in all 13 patients. Five out of 13 patients had predominant C3 deposits. IgM deposition was variable. Only four patients had C1q deposits in glomerular mesangium and basement membrane. IgG4-containing immune complexes were present in nine out of 11 histopathological specimens. Figures 3, 4 show immunofluorescence images.

These images showed positive granular IgG-containing membranous and IgA-containing mesangial immune deposits.

Patients received one out of five treatments and their response has been summarized in Table 4.

**Table 4:** Summary of treatment modality and response to therapy ACEi: angiotensin-converting enzyme inhibitors, ARB: angiotensin receptor blocker, CR: complete remission, PR: partial remission, NR: no remission, CNI: calcineurin inhibitor, MMF: mycophenolate mofetil

Case list	ACEi/ARBs, spironolactone	ACEi/ARBs, spironolactone, steroids	ACEi/ARBs, spironolactone, CNIs	ACEi/ARBs, spironolactone, steroids, CNIs	ACEi/ARBs, steroids, MMF	Response to therapy
Case 1		Yes				PR
Case 2				Yes		CR
Case 3		Yes				CR
Case 4			Yes			CR
Case 5				Yes		NR
Case 6	Yes					NR
Case 7				Yes		CR
Case 8	Yes					NR
Case 9					Yes	CR
Case 10				Yes		CR
Case 11	Yes					NR
Case 12				Yes		CR
Case 13	Yes					CR

As seen in the table, five out of 13 patients received ACEi/ARBs, spironolactone, steroids and CNIs. Out of these five patients, four patients achieved complete remission. Four out of 13 patients received only non-immunosuppressive, anti-proteinuric therapy that included ACEi/ARBs and spironolactone. Only one out of these four patients achieved complete remission. Two out of 13 patients received steroids in addition to non-immunosuppressive therapy. Out of these two patients, one achieved complete and other achieved partial remission. Remaining two patients received CNIs and MMF in addition to non-immunosuppressive therapy. Both these patients achieved complete remission. In short, eight out of 13 patients achieved complete remission.

## Discussion

In summary, this case series has briefly described major aspects of coexistent IgA and membranous nephropathy. According to our study, the overall prevalence of this coexistent disorder is around 1.75%. Among the 13 studied cases, female predominance of this coexistent disorder was noted (53.8%). This finding is in contrast to one study published in 2016 [1]. There was a wide age range starting from 14 to 71 years. Therefore, it is proposed that this coexistent disorder can present at any time irrespective of any particular age limit.

As mentioned, MN combined with IgAN comprises the clinical and pathological features of both diseases [4]. Our patients had nephrotic syndrome and a normal renal function, which is consistent with membranous nephropathy and microscopic hematuria that is the characteristic feature of IgAN. Similar observations have been published in other studies as well [7]. In contrast to the classical feature of IgAN, macroscopic hematuria was not reported in any of our patients.

The exact pathogenic mechanism of this coexistent disorder is not known [7,11]. Several theories have been published in the past. Kobayashi et al. considered that the coexistence of IgAN-MN did not occur due to chance alone, and should be regarded as an entity in glomerular pathology [12]. Frascà et al. reported that IgAN-MN occurred separately in one patient at an interval of seven years [13]. Miyazaki et al. reported that IgAN developed 14 years after a diagnosis of MN [10]. Another study postulated that the occurrence of superimposed MN on a background of preexisting mild IgAN caused combined IgAN-MN [7]. We agree with the last postulation as most of our patients had clinical and prominent histopathological findings of MN that might have led to their hospital presentation. However, no conclusion can be made as the renal biopsy showed findings of both disorders present at the same time in the same patient.

Regarding immunofluorescence findings, there were granular IgG deposits in the glomerular basement membrane and moderate to marked granular IgA deposits predominantly in the mesangial area. This classical finding of this overlapping disorder has been mentioned in several case reports [1,3,4,6,7]. In addition to this, a greater number of our patients had C3 deposits in comparison to C1q deposits. It has been postulated that IgA-containing immune complexes lead to C3 deposition and IgG-containing immune complexes lead to C1q deposition [14,15]. In other words, it is possibly indicated that there is greater and earlier activation of an alternate pathway in comparison to the classical pathway. Therefore, we suggest that on the background of IgA nephropathy, there might be occurrence of membranous nephropathy leading to more prominent features of membranous nephropathy later on, thus further supporting the mentioned preposition [7].

There are few case reports published that described the pathogenic role of hepatitis B antigen in the simultaneous development of IgAN-MN [16,17]. This observation has been negated by our study, as there was not a single patient who turned out to be hepatitis B positive during one's workup of nephrotic syndrome. However, one patient was hepatitis C positive (case 2) and one patient (case 9) had strong ANA positivity, who later turned out to be a case of lupus nephritis and was successfully treated with MMF and steroids. ANA positivity was also detected in three patients of a recently published clinical study, thus supporting this finding [7].

According to a recently published study, serum anti-PLA2R antibodies have been considered an increasingly important biomarker that can support in the diagnosis of membranous nephropathy associated with IgA nephropathy [4]. However, in our patients, anti-PLA2R antibodies were positive in only one and negative in seven patients in spite of IgG4 positivity on immunofluorescence. Therefore, it can be suggested that the prevalence of anti-PLA2R antibodies is much lower in the coexistent disorder in comparison to isolated membranous nephropathy.

On light microscopy, there was mesangial hyper-cellularity with mesangial expansion and glomerular basement membrane thickening. Most of our patients had lower MEST-C and TRCS scores. This supported that coexistent IgA and membranous nephropathy is associated with a lower MEST-C score than the isolated IgA nephropathy [7].

One case report published recently has mentioned successful treatment of this coexistent disorder with renin-angiotensin-aldosterone blockade, corticosteroids, and CNIs [4]. Four out of five patients, who received this therapy, underwent complete remission. One patient (case 5) had not responded to this treatment combination, most probably due to higher baseline serum creatinine, greater degree of glomerular sclerosis, presence of crescentic glomerular nephritis, higher overall MEST-C and TRCS scores indicating delay in presentation and diagnosis of this coexistent disorder. Therefore, we conclude that the treatment decision should be made according to renal biopsy findings. Besides this, the combination of CNIs, steroids and ACEi/ARB therapy is found to play an effective role in the management of this coexistent disorder. However, further studies are needed to validate these findings.

It also helps in providing guidance regarding the management of patients with the coexistent disorder based on the severity of histopathological features. This proposition is based on the finding that one of our patient (case 13) even responded completely to non-immunosuppressive therapy. This is because renal biopsy findings of this patient showed normal glomerular basement membrane thickening, minimal mesangial hyper cellularity, no glomerulosclerosis, tubular atrophy, interstitial fibrosis, 0 TRCS score and an MEST-C score of 1 only. The remaining three patients who were given conservative therapy had not responded possibly due to moderate to marked glomerular basement thickening on light microscopy. Therefore, it is proposed that the decision regarding starting either conservative or immunosuppressive therapy must be based on the degree of glomerular basement membranes thickness.

The results of previous case studies proposed that overlapping MN and IgAN appears to correlate with an increased incidence of disease remission [2,4,6,8]. This has been supported by our study findings in which eight out of 13 patients achieved complete remission. However, there are multiple factors that can alter the outcome that include time of presentation after the onset of symptoms, associated co-morbidities, histopathological severity and chronicity score and type of treatment received by the patient.

The major limitation of this study is the small sample population; therefore, results obtained from this study need further validation from studies of a large sample size.

## Conclusions

MN-IgAN is most likely a distinct clinicopathological disorder other than either membranous or IgA nephropathy. Immunofluorescence microscopy is the best diagnostic modality for this coexistent disorder. The serum PLA2R antibody positivity rate was lower in concurrent MN-IgAN patients. The complete remission rate was higher for this coexistent disorder indicating a better prognosis of the coexistent disorder than either IgA or membranous nephropathy alone. In addition to this, we conclude that the combination of CNIs, steroids and ACEi/ARBs is possibly the best modality of management of this coexistent disorder. However, further studies are needed to confirm these findings.
